# Isolated Renal Metastasis from Non-Small-Cell Lung Cancer: Report of 2 Cases

**DOI:** 10.1155/2015/357481

**Published:** 2015-11-04

**Authors:** Masaki Tomita, Takanori Ayabe, Eiichi Chosa, Kunihide Nakamura

**Affiliations:** Department of Surgery, Faculty of Medicine, University of Miyazaki, Kihara 5200, Kiyotake, Miyazaki 889-1692, Japan

## Abstract

Renal metastasis from non-small-cell lung cancer is rather uncommon; isolated metastasis especially is rare. Herein we report 2 cases who developed a solitary renal metastasis after undergoing a curative resection for non-small-cell lung cancer. They received nephrectomy.

## 1. Introduction

Renal metastasis from non-small-cell lung cancer (NSCLC) is commonly found in autopsy series [1, 2], but it is rarely diagnosed in live patients [2–4]. Isolated metastasis especially is rare [2–5].

Herein we report 2 cases of isolated renal metastasis from NSCLC. These cases also received nephrectomy.

## 2. Case Presentation

### 2.1. Case  1

**Figure 1:**
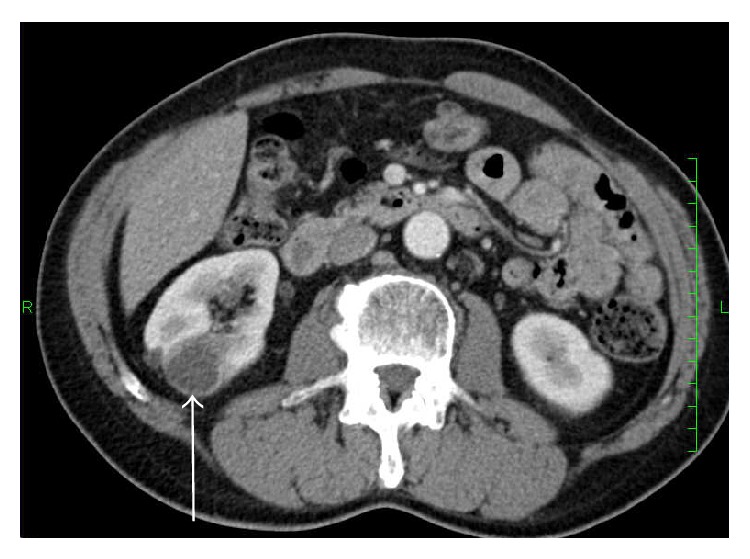
Computed tomography showing the isolated right renal mass (arrow).

**Figure 2:**
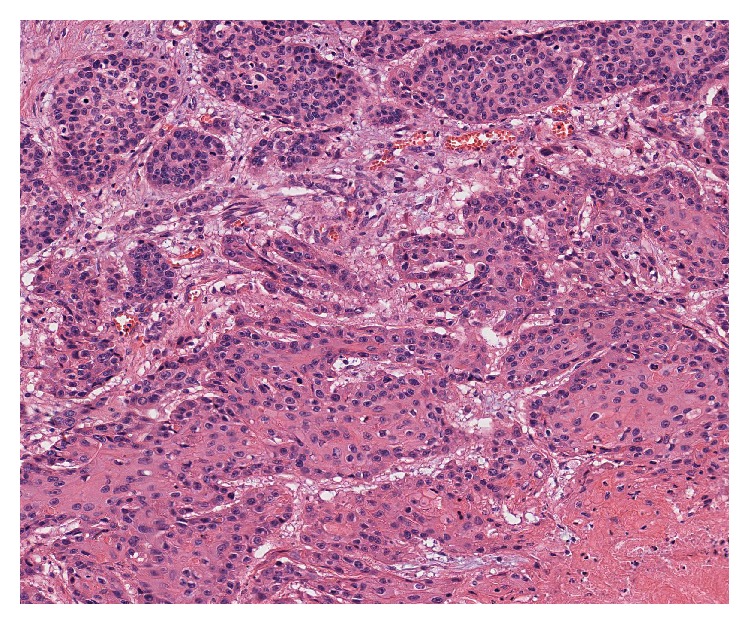
Histology of renal tumor of case 1 showing squamous cell carcinoma (H & E, ×100).

A 63-year-old Japanese man was admitted with squamous cell carcinoma of the lung. Then he had left pneumonectomy and lymph nodes exploration. Postoperative pathologic examination revealed moderately differentiated squamous cell carcinoma without lymph node metastasis (pT2N0M0). After 1 year and 6 months of disease-free interval, a routine enhanced computed tomography (CT) scan showed a hypodense mass (25 mm in the largest dimension) in the lower pole of the right kidney (Figure 1) although he had no hematuria or any other symptoms. The renal biopsy was performed and biopsy specimen showed a metastasis from NSCLC. Thus he received cisplatin (CDDP) and gemcitabine (GEM) combination chemotherapy (intravenous CDDP 70 mg/m^2^ on day 1 plus GEM 1000 mg/m^2^ on days 1 and 8) for 1 cycle. Due to renal function decline, subsequently he received 4 cycles of intravenous carboplatin (area under curve (AUC) = 5) on day 1 and GEM (1000 mg/m^2^) on days 1 and 8, every 3 weeks. During chemotherapy he sometimes had macroscopic hematuria, and no effect of chemotherapy was obtained. After chemotherapy, he underwent right nephrectomy for a renal metastasis. Pathological examination of the specimen led to a diagnosis of squamous cell carcinoma (Figure 2). Two years and 6 months after nephrectomy, the patient has been doing well, with no signs of recurrence.

### 2.2. Case  2

**Figure 3:**
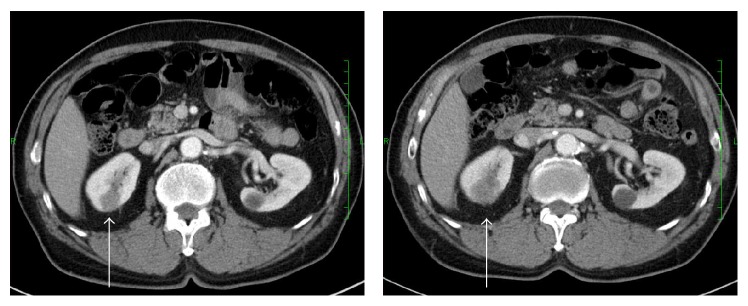
Computed tomogram of the abdomen showing right renal metastasis (arrow). The tumor increased in size during follow-up.

**Figure 4:**
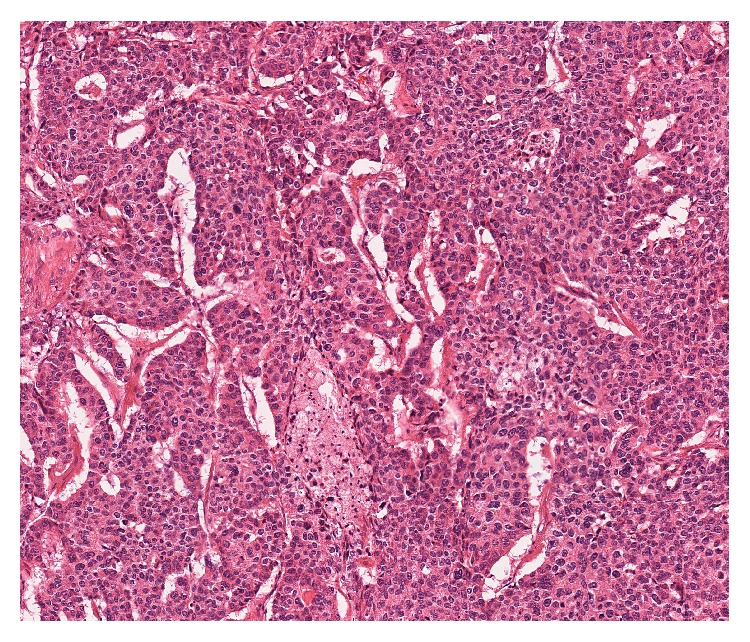
Histology of renal tumor of case 2 showed carcinoma cells arranged in nests, cords, or tubules. Immunohistochemically, the tumor cells are positive for TTF-1, napsin A, and surfactant apoprotein (not shown) (H & E, ×100).

A 65-year-old Japanese man received right upper lobectomy for right lung adenocarcinoma (pT1aN0M0). A histological examination showed adenocarcinoma of the lung and no metastatic foci in dissected lymph nodes. He received oral uracil-tegafur (250 mg of tegafur per square meter of body-surface area per day) for 2 years. During the regular postoperative follow-up as an outpatient, his serum carcinoembryonic antigen (CEA) level began to elevate gradually 4 years after surgery. He was asymptomatic, with no abdominal discomfort, hematuria, or any other urinary symptoms. Radiological examination, including chest CT, brain magnetic resonance imaging, and positron emission tomography scan failed to find the recurrence. However serum CEA level continued elevating. Three months after, we performed CT scan again. We detected a hypodense mass in the lower pole of the right kidney increasing in size (20 mm to 26 mm), which misdiagnosed as a renal cyst in the previous examination (Figure 3). There were no other visceral metastases or lymphadenopathies. He refused the chemotherapy and underwent a radical nephrectomy. A histological examination of the resected kidney showed a metastatic adenocarcinoma, which coincided with the histological findings of the previously resected NSCLC (Figure 4). His serum CEA level returned to within normal range after nephrectomy. He refused postoperative chemotherapy and he has been doing well without recurrences 1 year after nephrectomy.

## 3. Discussion

Since the renal blood flow accounts for approximately 20% of cardiac output, the kidneys are likely to be vulnerable to hematogenous metastases. Although renal metastases from NSCLC are frequent at postmortem examination [1, 2], clinically recognized isolated metastasis to the kidney from NSCLC is rare. Scatena et al. [5] reviewed that only 35 cases with renal metastatic lesions from lung cancer are reported to date in the English literature. They reported that renal localization of NSCLC is extraordinarily uncommon [5]. Majority of renal metastases are usually part of disseminated disease or bilateral renal metastases [6, 7]. Therefore isolated renal metastasis from NSCLC is rather uncommon.

In Japanese series, Ichimatsu et al. reviewed 64 cases of metastatic renal tumors originating from lung cancer reported in Japan [8]. Although this series contains patients with some bilateral renal metastases and 5.1% of small-cell lung cancer, the summary of this series is as follows [8]. The frequency of male gender was approximately 3 times more than that of female. About half of patients had hematuria and 18% of patients are asymptomatic. Histologic subtype was 57.6% of squamous cell carcinoma, 28.8% of adenocarcinoma, and 5.1% of small-cell carcinoma. 41/64 (66.1%) patients received nephrectomy. The 1-year survival was 19.2% in overall patients and 33.4% in patients who received nephrectomy.

Honda et al. [9] reported the CT findings of metastatic renal tumor. They concluded that renal metastases were characterized as small, multiple, bilateral, wedge-shaped, less exophytic, and located within the renal capsule [9]. Unfortunately, our cases did not have many of these characteristics. We misdiagnosed the metastatic lesion as renal cyst in case 2. Honda et al. [9] discussed that complicated cysts and hyperdense cysts might be confused to differentiate from metastasis although multiple cysts are easily distinguished. Voci et al. [10] revealed that delayed contrast enhanced CT is useful to distinguish renal cell carcinomas and nonneoplastic cysts. Further studies in this area are warranted.

The role of solitary nephrectomy from NSCLC has been unknown in detail because comparison of surgical versus nonsurgical modalities in the management of solitary metastatic disease in prospective randomized studies seems to be very difficult. Karagkiouzis et al. [11] review the possible role of surgical resection in the management of NSCLC with solitary metastasis. They concluded that surgical resection as part of an aggressive approach positively affects patients' survival in highly selected patients with isolated metastasis [11]. Factors related to the favor outcome were reported to be control of primary site, confirmed solitary metastatic disease, good performance status, metachronous lesions, and longer disease-free interval [11]. Therefore nephrectomy might be one of useful options in selected NSCLC patients with isolated renal metastasis.

## 4. Conclusion

Renal metastasis should be considered whenever a mass in the kidney is identified in patients with a prior history of resected localized NSCLC. Although the prognosis is poor even when surgery is performed, we believe that nephrectomy might be one of useful options in the absence of disseminated disease or in selected patients. Further evaluation should consider this hypothesis.
